# Development of a multiple-hybrid population for genome-wide association studies: theoretical consideration and genetic mapping of flowering traits in maize

**DOI:** 10.1038/srep40239

**Published:** 2017-01-10

**Authors:** Hui Wang, Cheng Xu, Xiaogang Liu, Zifeng Guo, Xiaojie Xu, Shanhong Wang, Chuanxiao Xie, Wen-Xue Li, Cheng Zou, Yunbi Xu

**Affiliations:** 1 Institute of Crop Science, National Key Facility of Crop Gene Resources and Genetic Improvement, Chinese Academy of Agricultural Sciences, Beijing 100081, China; 2 International Maize and Wheat Improvement Center (CIMMYT), El Batán 56130, Texcoco, Mexico

## Abstract

Various types of populations have been used in genetics, genomics and crop improvement, including bi- and multi-parental populations and natural ones. The latter has been widely used in genome-wide association study (GWAS). However, inbred-based GWAS cannot be used to reveal the mechanisms involved in hybrid performance. We developed a novel maize population, multiple-hybrid population (MHP), consisting of 724 hybrids produced using 28 temperate and 23 tropical inbreds. The hybrids can be divided into three subpopulations, two diallels and NC (North Carolina Design) II. Significant genetic differences were identified among parents, hybrids and heterotic groups. A cluster analysis revealed heterotic groups existing in the parental lines and the results showed that MHPs are well suitable for GWAS in hybrid crops. MHP-based GWAS was performed using 55 K SNP array for flowering time traits, days to tassel, days to silk, days to anthesis and anthesis-silking interval. Two independent methods, PEPIS developed for hybrids and TASSEL software designed for inbred line populations, revealed highly consistent results with five overlapping chromosomal regions identified and used for discovery of candidate genes and quantitative trait nucleotides. Our results indicate that MHPs are powerful in GWAS for hybrid-related traits with great potential applications in the molecular breeding era.

Genetic mapping of important agronomic traits, followed by marker-assisted selection (MAS), provides a powerful tool for crop genetic improvement. Genes can be mapped through four basic approaches: linkage analysis using bi- or multi-parental populations, association or linkage disequilibrium (LD) analysis using natural populations, comparative analysis using mutated populations and near-isogenic (introgression) lines, and selective analysis using sub-populations based on selective sweeps.

Association mapping has been used to detect the underlying major genes in the gene pools and their introgression to improve traits in major crop breeding programs^1^. It has been based on two basic methods, one using candidate gene-based markers to confirm the association^2^ and the other using whole genome scan^3^, the latter being called genome wide association studies (GWAS). GWAS using single nucleotide polymorphism (SNP) marker loci has successfully identified genes and pathways for agronomic traits in many crops of economic importance, including rice^4^, maize^5^, wheat^6^, sorghum^7^ and barley^8^. This method generally consists of five stages: selection of diverse germplasm, estimation of the level of population structure, phenotypic evaluation, genotyping for candidate genes or whole genome genotyping, and statistical test for genotype-phenotype association^9^.

In contrast to linkage mapping, GWAS based linkage disequilibrium (LD) offers a potentially useful and robust approach for mapping causal genes with moderate or large effects^10^, which has several advantages: extensive genetic variations in a more representative genetic background, higher resolution, and utilization of historic phenotypic data on cultivars without the need to develop special mapping populations^11^. The simple statistical model for GWAS is focusing on single-SNP tests, and the test results frequently show high false positives owing to specific problems such as population structure, relatedness and polygenic background effects. Therefore, a variety of statistical analytical methods have been developed, such as, the mixed linear model (Q + K model), which is the most popular method that effectively eliminates false positives by incorporating population structure (Q) and relative kinship matrix (K)^12^, multi-trait mixed model (MTMM) for multiple traits^13^, multi-locus mixed-model (MLMM) based on multiple loci^14^, factored spectrally transformed linear mixed model (FaST-LMM) with the number and square of rank of the relationship among individuals^15^, settlement of MLM under progressively exclusive relationship (SUPER) using influential bin markers and a small set of markers to define the relationship among the individuals^16^, multi-trait set linear mixed-model (mtSet-LMM) between sets of variants and multiple traits^17^, and a random-SNP-effect MLM (RMLM) with a modified Bonferroni correction and a multi-locus model with less rigorous selection criteria from RMLM (MRMLM)^18^.

There are many types of populations that have been used in genetics, genomics and crop improvement^19^,^20^. These populations have been used individually, and in very few cases, in combination. The primary objective for this article was to review all available populations, and introduce the concept of multiple-hybrid population (MHP) as a new population type, which is more suitable for GWAS in hybrid crops using hybrid vigor. Using maize as an example, we developed an MHP from diallel and NC II mating designs. We will present the experimental design, parent classification, data analysis strategies and applications of the MHP in conventional quantitative genetics, GWAS, and breeding.

## Populations in Genetics, Genomics and Crop Improvement

Populations used in genetics, genomics and crop improvement have their advantages and disadvantages. For the convenience of comparison, we will first discuss all available populations and then focus on the MHP in the next section.

### Biparental populations

The populations derived from two parental lines are most widely used in genetics, genomics and breeding, with the following advantages and disadvantages:

- F_2_: containing sufficient genetic information, but failing to distinguish the genotypes just by phenotyping owing to the presence of heterozygotes, and thus the phenotyping is usually measured with its derived F_2:3_ families, progenies produced by bulked pollination of F_2_ individuals, and the F_2_ genotypes maintained by multiple tillers and ratoning procedures^19^,^20^.
- BC_1_: including two types of genotypes which could be distinguished theoretically just by phenotyping if the target gene is dominant, but difficult to maintain the materials for long time, although the genotypes may be maintained by following the procedures for F_2_ individuals as discussed above.
- RIL (recombinant inbred line): consisting of genetically stable genotypes, which can be maintained permanently without change of the population constitution, and suitable for distinguishing dominant and co-dominant statuses, but difficult to be bred for some species, especially for cross-pollinated plants^21^,^22^.
- DH (doubled haploid): including homogeneous and breeding-true genotypes and reflecting the gene segregation and recombination rate of F_1_ gametes, with different protocols available for the development of DH lines including microspore culture, chromosome elimination, and haploid inducers, depending on crop species^19^,^20^.

An immortalized F_2_ population can be developed through crosses among RIL or DH populations, which can be used permanently for QTL analysis^20^,^23^.

### Multiparental populations

There are three major types of multi-parental populations, four-way crosses (4WC), NAM (nested association population) and MAGIC (multiparent advanced generation inter-cross). The 4WC were widely used in commercial animal and plant breeding, formed by a cross between two hybrids, F_1_ (P1 × P2) and F_1_’(P3 × P4), by which genes and QTL can be identified^24^ with epistatic QTL mapping developed using penalized maximum likelihood (PML)^25^.

NAM population is developed by crossing a reference genotype (line) with a panel of lines maximizing the genetic diversity and RIL families are then developed. Different sets of RIL populations share a common parental line, while RILs within each population are derived from two parents. The NAM population combines the advantages of both bi-parental segregating and natural populations, which can be used for integrated linkage and association mapping^26^. The first NAM population in plants was developed by using 200 RILs from each of the 25 crosses derived from 25 diverse inbred lines crossed with a reference inbred line B73, which resulted in nearly 5000 RILs in total^27^. This NAM population has been successfully applied to the study of flowering time^28^, resistance to southern corn leaf blight^29^, leaf architecture^30^ and carbon and nitrogen metabolism^31^. However, 48.7% of marker genotypes were from B73 among these RILs and allelic frequencies from common parent are much higher than those from other 25parents with phenotype deviation due to the only one common parent.

The MAGIC population starts with multiple bi-parental intercrosses based on variety-specific founders selected, and then every two bi-parental F_1_s are intermated to produce a double hybrid. The two double hybrid F_1_s are intermated again to develop a hybrid with eight parents involved^20^. This process continues until all parental lines are included in a final hybrid, and then RILs can be developed by selfing at least five generations or DHs generated through an available protocol^32^. The first MAGIC population has been developed in *Arabidopsis thaliana* by using a set of 527 RILs descended from a heterogeneous stock of 19 intermated accessions^32^. MAGIC populations have been developed to significantly increase the effectiveness of whole genome scan in wheat^33^, rice^34^ and maize^35^.

### Mating-design populations

There are several populations that can be derived from specific mating designs, including:

- Diallel: multiple parents are crossed to produce all possible bi-parental F_1_s.
- North Carolina Design (NC) I: some individuals randomly selected as males from a biparental F_2_ population are crossed with other randomly selected as females.
- NC II: *n* parental lines are divided into two groups, one group as males and the other as females, to produce hybrids of all possible combinations.
- NC III: *n* individuals are selected from an F_2_ population to backcross its parents, P_1_ and P_2_.
- TTC (triple test crosses): an extension of NC III, i.e., *n* individuals (*n* > 20) are selected from an F_2_ population to backcross with both parental lines, P_1_ and P_2_, and their F_1_s.
- sTTC (simplified triple testcrosses): *n* cultivars or strains are selected from the germplasm pool to cross with two varieties or strains, P_H_ and P_L_, with the highest and lowest phenotypic values.

### Natural populations

The natural populations are those consisting of many individuals that are fixed or can be maintained individually through selfing or outcrossing, including inbred lines, varieties, landraces and wild relatives, which have been widely used in association mapping. There are two types of natural populations. The natural populations composed of elite lines have lower diversity and slower LD decay with a longer distance, while those with a core collection of diverse germplasm have rich genetic diversity and more rapid LD decay with a shorter distance. Both types can be combined in applications. First, rough mapping for a particular trait can be performed with the genetic populations with lower diversity and a small amount of markers to cover the whole genome; then, using natural populations with rich diversity in the genomic region of interest, a particular gene can be fine mapped for gene cloning^36^.

## Multiple-Hybrid Populations (MHP) for GWAS

**Figure 1:**
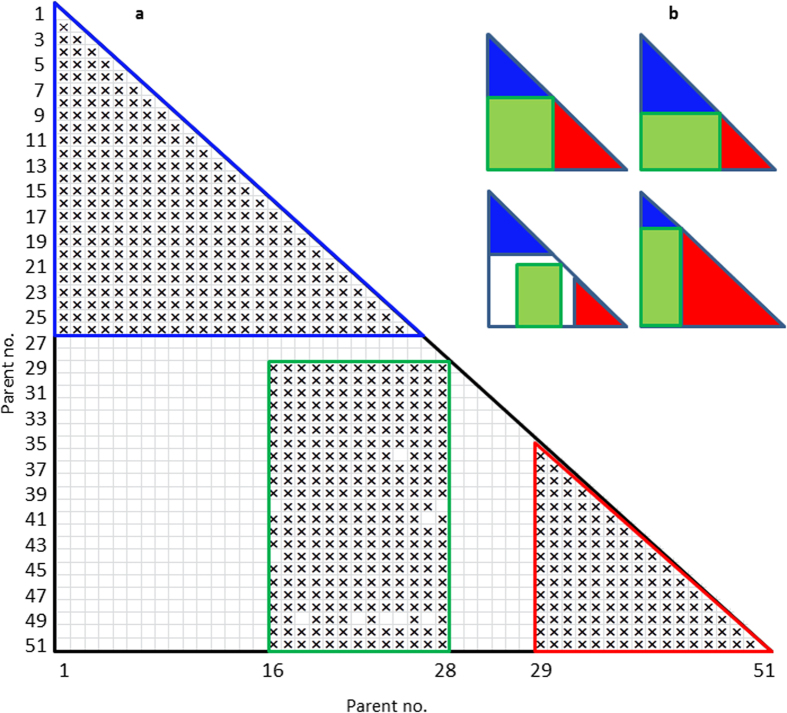
Development of MHP with diallel and NC II mating designs. (**a**) An MHP developed in this study with two sets of diallel hybrids, one from 26 temperate maize inbred lines (blue triangle, P1-26) and the other from 17 tropical maize inbred lines (red triangle, P35-51), and one set of NC II hybrids (green box) with 13 temperate (P16-28) by 21 tropical inbred lines (P29-51). See Table 1 for the parent numbers and names. The 724 maize hybrids made in this study can be used to predict the missing hybrids (empty boxes) and the rest hybrids (empty areas) that can be made from all the parents (any of the 1275 hybrids that can be derived from the full diallel set with 51 parents). (**b**) A full diallel (largest triangle) can be divided into three subpopulations (including two diallels and one NC II). Depending on the numbers of parental lines from different ecotypes, the three subpopulations, represented by blue, green and red, may vary to optimize crossing designs. The maize MHP developed in this study is shown on the bottom left.

Populations described above, except for those derived from various mating designs, are most appropriate for crops using inbreds or varieties for commercialization but not suitable for hybrid crops that use hybrids for production, as genetic researches based on inbreds reveal neither what are hidden in hybrids nor what are associated with hybrid performance. Populations for hybrid crops and hybrid performance may come from two sources, one with testcrosses using all individuals from each of the populations as parents to testcross with one or several testers, and the other with a large number of multiple hybrids generated from such as mating designs described in the previous section as a population, which can be simply called as multiple hybrid population (MHP). Considering all available mating designs that have been used in quantitative genetics, hybrids from a full diallel design with *n* parents would be one of the best MHPs, as the full set hybrids can be divided into three subsets, two diallels and one NC II, as needed. As such an attempt with a limited number of hybrids, a partial NC II was used for QTL mapping to test effects associated with traits, and the significant effects identified by empirical Bayesian were used to estimate genetic effects for the missing F_1_s with elite parents and hybrids predicted^37^. The generated hybrids, along with the parental genotype and phenotype, can be used to predict the missing hybrids and the rest hybrids. The relative sizes of the three subsets can be optimized to best match different ecotypes of parental lines, and the number of hybrids can be optimized so that a best design can be achieved with the smallest sample sizes as possible for the largest “genetic gain” (Fig. 1). As an example, we developed an MHP with hybrids from Griffing IV diallel crosses and NC II design using temperate and tropical elite maize inbreds as parental lines.

The parents used for developing MHP can be a representative sample of the population to which inference is desired and a core collection from a gene bank, varieties or landraces representing the elite germplasm for a breeding program, or a set of inbred lines representing a synthetic outcrossing population. MHPs have several distinct characteristics that make them very unique compared to other types of populations and very useful in genetics and plant breeding.

### Suitability for combining ability and heterosis analysis

The evaluation of hybrids produced by inbred lines is an important step towards the development of hybrid varieties in crops such as maize and this process theoretically should be done for all possible ways of hybridization (diallel crosses), where selection of favorable traits for each inbred can be determined. The diallel and NC II design analysis can provide elaborate information on the genetic identity of genotypes especially on dominance-recessiveness relationships and some genetic interactions^38^. With diallel or NC designs, many studies have been conducted for combining ability and heterosis analysis. For example, 30 crosses developed in maize with six parents according to Griffing III method to identify hybrids expressing a high level of heterosis^39^. The overall increase of GCA and SCA was found in NC II design between some common testers and 25 mutant lines from SP_4_ derived from three inbreds carried into satellite, providing useful information for maize breeding through mutation^40^. A Combined Relative Level (CRL) model using 112 metabolite levels in young roots from four parental inbred lines and their diallel hybrids in maize indicates that parental metabolite profiles can be used together with selected hybrids as a training set to predict biomass of all possible hybrids^41^. A complete diallel series of crosses involving eight parents was developed to evaluate GCA and SCA for corn grits^42^. A partial NC II design for cotton and rapeseed using epistatic association mapping revealed additive and additive by additive interaction effects for GCA, and dominance related effects for SCA. Mid-parent heterosis, dominance and dominance by dominance interaction effects affect heterosis more than over-dominance and complete dominance^43^. The MHP developed in our research will be used to estimate combining ability, heterosis, and genotype by environment interaction and details will be reported elsewhere.

### Easy for sharing through parental lines

Compared to natural populations used for GWAS, which needs to share at least several hundreds, even thousands, of varieties, MHP just needs to maintain and share a much less number of samples. Sharing germplasm accessions that can be used as parental lines is more frequent for germplasm exchange. For *n* varieties, *n(n*−1)/2 hybrids can be produced. For example, 100 varieties can be used to generate 99 × 100/2 = 4500 hybrids. It would be much more difficult to share 4500 samples compared with sharing 100 parental lines. In our study, the MHP, consists of 724 hybrids, generated through just crossing 51 maize inbreds with each other (Fig. 1), which is time- and labor-saving.

### Flexibility for testing across diverse environments

Diverse germplasm including inbreds and hybrids can be further investigated to identify new sources of genetic variations for fundamental experiments and commercial breeding. MHPs provide an opportunity for testing across diverse environments because of their better adaptation compared to inbred populations. The most typical example is the large-scale yield trials and multi-location tests before commercialization. Testing of multiple hybrids across diverse environments has been also used in genetic study. For example, testcrosses between Tx714 and 346 diverse inbreds evaluated under well watered and non-irrigated trials showed high genetic variance, and for 10 quantitative trait variants for agronomic traits as revealed by 60,000 SNPs, three of them explained 5–10% of phenotypic variation in grain yield under both water conditions^44^. In our research, hybrids from the MHP showed a high level of genetic and phenotypic variation across different environments (data not shown).

Through multi-environmental trials, adapted hybrids can be identified for specific environments. The difficulty of choosing appropriate varieties has contributed to restricted breeding progress for biotic and abiotic stress tolerance in highly variable target environments. Hybrids produced from diverse inbreds will show significantly different responses to diverse environments and thus the hybrids most adapted to a specific environment can be identified and used for further testing and breeding. In this regard, 91 hybrids made among seven high- and seven low-Fe-Zn content lines were evaluated in six locations, and one low-Fe-Zn parental line showed a significant positive GCA effect and its hybrids emerged as a highly promising variety^45^. A total of 84 hybrids were investigated for their resistance to parasitic weed *Striga* under artificial infestation and free environments in two locations, and three inbreds with high GCA effect for yield and *Striga* resistance and four high yielding hybrids showed the potential for further use^46^. In our MHP, some elite hybrids, especially large-scale commercialized ones, showed stronger adaptation under various environmental conditions.

### Suitability for both hybrid and inbred crops

Progeny selection is one of the most important stages in plant breeding. Crossing patterns and hybrids as those generated in MHP are commonly used in both hybrid and inbred breeding programs. For hybrid crops, the crosses are usually made among heterotic groups to achieve a high level of heterosis and better adaptation to specific locations and crop seasons. A total of 91 hybrids, evaluated along with their parental lines for combining ability and heterotic pattern, showed extent differentiation for different yield traits, and three best heterotic patterns identified were potentially useful for commercial maize breeding^47^. In our MHP, diallel and NC II crosses made between temperate and tropical elite inbreds were used to explore the genetic factors associated with combining ability and heterosis for hybrids development. MHP can be also used in self-pollinated species, although production of hybrids is much more difficult than outcrossing species. There are numerous available examples using diallel crosses in self-pollinated crops. For example, non-additive gene effect for most of yield components was revealed based on 28 rice hybrids from a partial diallel crossing of eight inbreds, five of which showed significant favorable SCA effect on yield^48^. Earlier stem elongation in floating rice can greatly improve the chance of floating rice to survive in the flood, and therefore a set of 6 × 6 half-diallel crosses with four floating and two non-floating rice varieties was developed with results indicating that the additive effect was higher than dominant effects and the dominant alleles were concentrated in the floating parental lines^49^. A partial diallel based on six *indica* and seven *japonica* rice genotypes was used to investigate the genetic variations of yield and cold tolerance, with significant heterosis and combining ability revealed for tested characters^50^.

### Savings in genotyping

As only the parental lines need to be genotyped, from which hybrid genotypes can be inferred, using MHP can save great investment in genotyping, compared to genotyping the same number of inbreds. As we calculated in the precious section, genotypes for 4,500 hybrids can be inferred from 100 parental lines genotyped, the former being 45 folds larger. In *Brassica napus*, 205 SSR markers were used to examine the polymorphisms among 441 parents (298 sterile and 143 restorer lines), and the genotypes for the partial NC II hybrids could be then deduced from their parents^37^, with 8 main-effect and 37 interacted QTL identified for oil content, which could be used to predict 10 elite restorer lines, 10 elite sterile lines and 10 elite hybrids.

## A Maize Multiple Hybrid Population

### An MHP from diallel and NC II designs

A total of 51 maize inbred lines, representing a broad selection of breeding germplasm from temperate and tropical regions, were used for development of an MHP (Fig. 1; Table 1). These included 28 temperate and 23 tropical inbred lines as parents, by which three subpopulations of MHP were developed: (1) temperate diallel consisting of 325 crosses made in Griffing IV using 26 elite maize inbred lines, including 13 U.S. and 13 Chinese inbred lines that represent different heterotic groups; (2) temperate and tropical NC II consisting of 263 hybrids made between 13 temperate and 21 tropical inbred lines; (3) tropical diallel consisting of 136 crosses made in Griffing IV using 17 tropical inbred lines as parents, most of which come from CIMMYT.

### Chinese temperate inbreds used as MHP parents

Among 15 Chinese temperate inbreds used to develop the MHP (Table 2), Ye478, HZ4 (Huangzao 4), Dan340, Mo17, Tie7922 and Qi319 have been used as common tester lines for six Chinese heterotic groups, Reid, SPT, LRC, Lancaster, PA and PB, respectively^51^. These key inbreds have been playing a very important role in development of both inbreds and hybrids across maize regions in China. For example, Chang7-2 and LX9801 are among lines derived from HZ4 in summer maize region, Huang-Huai-Hai River Zone^52^. A total of 20 commercial hybrids have been developed by using Dan340-derived lines as parents, and 17 elite inbreds have been developed using Dan340’s sister lines^53^. As a common tester line for heterotic group ‘SPT’, HZ4 has been used to develop more than 40 hybrids and nearly 70 elite inbreds, including Ji853, LX9801 and Chang7-2^54^. H21, an inbred line with high drought tolerance derived from Pioneer hybrid P78599^55^, and two superior hybrids made directly were planted widely in Huang-Huai-Hai River Zone. Four elite hybrids widely used in production were developed by Nan21-3, which was developed by selfing the elite exotic hybrids^56^. Qi205, a quality protein maize (QPM), was a breakthrough progress in protein maize in China^57^. More than 50 varieties, including Ludan50, Ludan981 and Ludan963, were released with Qi319, highly resistant to southern corn rust, and its derived lines as parents^58^. Over twenty inbreds were derived from Si287 with two widely used hybrids, Jidan27and Jidan46. Developed by seven continuous selfings from a U.S. hybrid 3382, Tie 7922 was the parent for 30 hybrids and numerous inbreds, including TieC8605-2, Dan9046 and Liao2345^59^. Ye478 has been used to produce nearly 50 hybrids, such as Zhongdan8, Yedan12, Yedan13, and Yedan19. Zheng22, belonging to the heterotic group LRC, was used in Yuyu18, a hybrid widely used in production. Zheng58 is the female parent of ‘Zhengdan958’, which had been planted over 4 million ha per year in three consecutive years^60^. Dan598, developed from Pioneer hybrid P78599 from tropical zone, was used to make several widely planted hybrids, mainly Danke series^61^. Huang C was the male for one of the best hybrids, Nongda108, which was awarded the First-Class Prize for China National Science and Technology Progress in 2002^54^.

### Tropical inbreds used as MHP parents

Tropical germplasm has abundant genetic variability and special resistance to diseases, pests and abiotic stresses. Introgression of favorable genes from tropical maize can broaden the genetic basis of temperate maize, improve abiotic and biotic stress tolerance, optimize heterotic patterns, and develop improved temperate-tropical hybrids. There are two primary ways for utilization of tropical germplasm in temperate maize breeding. One way is to conduct adaptive selection for tropical germplasm in transition regions, development of temperate-tropical composite populations and reciprocal recurrent selection for performance of temperate by tropical hybrids. Another method is to directly modify tropical maize that do not adapt in temperate conditions by selection for precocious, short flowering interval, and disease and lodging resistance. From the 1960s to early 1980s, U.S. scientists made great efforts for utilization of tropical maize. With more than 20,000 varieties of tropical maize collected^62^, they found that maize inbreds containing tropical germplasm were not only a useful source to expand the genetic diversity of temperate inbreds, but also competitive in crosses with temperate materials, producing high yielding hybrids^63^. For example, temperate by tropical hybrids showed greater adaption than temperate hybrids under heat stress environment^64^. In China, some tropical germplasm, such as Tuxpeno, Suwan and Mohuang9, were introduced in 1980s, with several excellent inbreds developed, such as 8703, S37, Taixi113 and 8501. In our MHP, 23 tropical inbred lines widely used in breeding programs of China and CIMMYT were included as parental lines, and our results also showed that the tropical lines had great potential for improvement of yield traits for temperate maize. Among three Chinese tropical inbred lines, Jiao51 was selected from landrace ‘Jiaomaerhuangzao’ in Guizhou, by which three elite hybrids, Jiaosandanjiao, Andan136 and Yudan11, were released^65^. Chuan 29 Female was selected based on natural pollination seeds from a single cross using pedigree method, with hybrid Chuandan29 released^66^. 18–599 was an elite inbred line in southwest China, with three elite hybrids, Shengyu9, Dong315 and Chunxi11, released for planting in mountain and deep hillock regions.

### Temperate by tropical hybrids in maize production

Maize exhibits an astounding capacity for environmental adaptation with wide distribution around the world, but genetic bottlenecks resulting from natural and artificial selection for adaptation and productivity lead to the differentiation between temperate and tropical maize. Improving the adaptability of temperate maize largely depends upon the use of tropical maize germplasm, which hosts rich sources of favorable genes and alleles^67^, particularly for tolerance to abiotic and biotic stresses. A shortcut to the favorable alleles hosting in tropical maize is to develop temperate-tropical hybrids. Considering the significant differentiation between the two groups, the best way to test temperate-tropical crosses is by NC II, where a set of temperate inbreds are used as parents to cross with a set of tropical inbreds.

Moving maize from tropical to temperate means the selection for adaptation to changing day length and temperature conditions. Such photo-thermal responses have been investigated intensively. An improved population BS16 was developed by selective adaption for ETO (developed at Estacion Tulio Ospina), with the characteristic of 21 days earlier in silking, increasing yield and combining ability^68^. Through GEM (Germplasm Enhancement of Maize) program that targeted at moving tropical lines into breeding program, six tropical lines were released^69^. A collection of 152 tropical populations were used to investigate photoperiod sensitivity under long-day conditions, showing that the highland populations displayed a weak photoperiod sensitivity^70^. Longer leaf stay-green has been considered as a visible character of temperate by tropical hybrids, and the ratio of visible source leaves (RSL) showed significant correlation with grain yield. As a result, RSL can be used as a selection criterion for improving stay-green and yield traits in maize breeding programs^71^.

Because of the maize responses to photoperiod and temperature regimes, typical temperate or tropical maize hybrids have limited growing seasons or zones. However, temperate-tropical hybrids, which combine genetic merits from both ecotypes, show much stronger adaptation to environmental conditions. As a result, depending on the relative genetic contribution from two parental lines, some temperate-tropical hybrids may be planted in wider maize production areas from temperate to tropical zones, while others may only adapt to typical temperate, subtropical or typical tropical regions. A previous research indicated that temperate-tropical hybrids could adapt to more diverse growing environments than temperate-temperate or tropical-tropical hybrids^72^. A biofuel potential investigation with 12 temperate-tropical maize and two grain and silage hybrids indicated that temperate-tropical hybrids showed more stalk biomass and 50% more sugar with supplemental fertilizer N, and could produce equivalent ethanol with a small amount of nitrogen fertilizer^73^.

### Agronomic performance of parental lines and their hybrids

Agronomic traits for inbreds and hybrids included in our MHP were evaluated for three years (2013–2015) in two locations (Beijing and Henan) with randomized block design each with two replications. Phenotypic performance across environments was evaluated using the best linear unbiased predictions (BLUPs)^74^ estimated by SAS PROC MIXED procedure with genotype, environment and year as random effects (Table 3). Compared to temperate parental lines, tropical inbreds showed higher plant and ear heights, delayed growth and development with significant larger days to tassel, silk and anthesis. For yield traits, however, temperate inbreds were overall better than tropical lines with higher grain number, row number, hundred grain weight and grain weight per plant. Among three hybrid subpopulations, significant delayed flowering time were found in both NC II and tropical diallel hybrids, and greater flowering time interval, anthesis-silking interval, was shown in NC II hybrids. For yield traits, NC II crosses showed higher ear length, ear diameter and hundred grain weight, while temperate diallels had greater grain number.

The 28 temperate inbreds used in the MHP could be divided into six heterotic groups, plus four Pioneer germplasms as an independent group (Fig. 1; Table 4). The hybrids from Lancaster parental lines showed the best performance for grain number per row, while the hybrids from PB parents showed the highest plant and ear heights, grain weight per plant, hundred grain weight and delayed flowering with a heterotic group-representative line, Dan340. Reid-derived hybrids had the shortest days to tassel while Pioneer hybrids had the shortest days to silk and anthesis. LRC hybrids showed lower ear height and greater row number and grain number, compared with other groups. Overall, PB germplasm have an edge on increasing yield, Pioneer germplasm can shorten flowering time, and LRC parents can be used to achieve high yielding by increasing grain number.

In conclusion, tropical germplasm can be used to improve the yield potential for temperate lines, while temperate germplasm, such as those from the U.S., are important donors to broaden genetic basis for specific breeding programs. As high genetic diversity and great trait variation were observed among parental lines and hybrids, the MHP developed in this study can be used in GWAS for many important traits. With known pedigrees and genotypic information from high-density markers, the structure and relationship among parental lines and thus among the hybrids can be well estimated and thus controlled in GWAS.

### Genotypic information generated for the MHP

The 51 parental lines used to develop the MHP have been genotyped by a newly developed maize 55K-SNP chip^75^. The chip contains 30,133 SNPs selected from 600 K Affymetrix^®^ Axiom^®^ Maize Genotyping Array, which evenly distributed on the genome, 4,049 high polymorphic SNPs from widely used 56K-SNP chip MaizeSNP50, 9,395 SNPs from whole genome RNA-seq^76^, 4,067 SNPs that are tropical specific and generated by resequencing to fill the gaps in the B73 reference genome, and 132 SNPs from the tags for published transgenic events. Genotyping the parents with the improved 55K-SNP chip will benefit genetic and breeding studies because of the improved genome coverage, compared to other SNP chips available.

## Applications of the MHP with diallel and NC II mating designs

**Figure 2:**
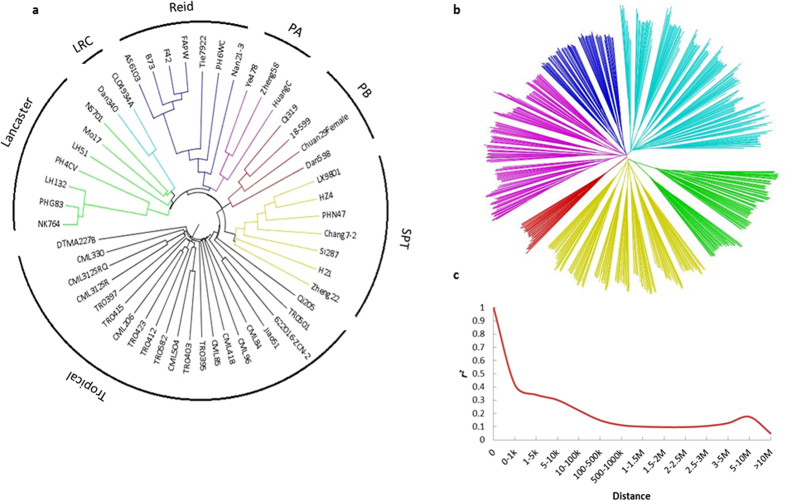
Neighbor-joining tree for the 51 parental inbreds and 724 hybrids (maize multiple-hybrid population, MHP) and LD decay across the whole genome. (**a**) The full tree separated the 51 maize inbreds into two major groups based on Roger’s genetic distance. The temperate inbred lines can be further divided into subpopulations corresponding to six Chinese maize heterotic groups. The tropical inbred lines showed a more complex genetic relationship with no definitive subgrouping. (**b**) The NJ tree for 724 hybrids was used to determine *K* of population structure for MHP. (**c**) Genome wide average LD for the MHP.

The MHP proposed in this article can be used to estimate combining ability and heterosis for yield and yield components using hybrid phenotypes compared with their parents, which can be further analyzed in GWAS along with other traits such as hybrid phenotypes *per se*. Associated information can be used for genome-wide prediction of hybrid performance. The MHP and its derived secondary populations can be also used as populations in genetics, genomics and plant breeding. As basic information required for applications of the MHP, a cluster analysis was performed to classify the parental lines (Fig. 2a). A neighbor-joining tree was constructed based on Roger’s genetic distance^77^, which clearly separated the parental lines into two major groups, temperate and tropical. The 28 temperate inbred lines were further divided into six heterotic groups, Lancaster, LRC, Reid, PA, SPT and PB, as revealed in previous studies^78^,^79^,^80^.

### Genetic analysis of combining ability and hybrid performance

Evaluation of combining ability and heterosis is the first step for breeding inbred lines towards the development of commercial hybrids. This process theoretically should go through for all parental lines and their possible hybrids. Both diallel and NC II designs can provide elaborate information on the genetic identity of genotypes, especially on dominance-recessive relationships and some genetic interactions^38^. As an example, a complete diallel crosses involving eight parents was produced to evaluate GCA and SCA for corn grits^41^. The MHP used in this research can be used to understand combining ability, heterosis, hybrid performance and genotype × environment interaction. This large-scale diallel-NC II design not only increases the power of statistical analysis, but also improves combining ability-based breeding efficiency for both temperate and tropical maize.

### GWAS based on markers, alleles and haplotypes

Genotyping of parental lines and then inferring their hybrid genotypes can be done through various genotyping platforms including sequencing (both whole genome and simplified genome sequencing or genotyping by sequencing, GBS) and chip-based genotyping. After genotyping, various markers such as SNP, copy number variations (CNV) and structure variation (SV), and their alleles and combinations (haplotypes) can be developed to cover the whole genome. For example, haplotypes have been used to construct HapMaps in maize with three different versions of HapMap developed^81^,^82^,^83^. RNA-seq and other sequencing technologies such as methylation-based sequencing have been used to identify expressed genes, epialleles and haplotypes associated with epigenetics^84^,^85^. Resequencing and GBS have been widely used in maize germplasm fingerprinting^86^, genetic diversity analysis^87^ and GWAS^88^.

Using numerous markers identified through various sequencing technologies, high density chips have been developed. In maize there are several high-density SNP chips available, including 56 K SNP chip^89^, 600K-SNP chip^90^ and a new 55K-SNP chip with improved genome coverage^75^. In human, high-density chips containing SV markers have been also developed^91^. These high-density chips can be updated with functional- and gene-markers so that more gene-related information can be generated. High-density chips provide a quick approach to reveal alleles, genotypes, haplotypes for a large number of samples with fixed and comparable markers. They have been widely used in maize germplasm fingerprinting^27^, genetic diversity analysis^80^ and GWAS^92^.

The currently available studies are mostly limited in a single application of genotyping methods, each with specific disadvantages. For example, gene arrays can not determine accurate positions of the target genes in multiple cell type, GBS may generate large quantities of missing data, and whole-genome resequencing shows high coverage but poor pertinence versus targeted resequencing. Therefore, simultaneous use of multiple genotyping techniques might be imperative. In soybean, 1.4 million tag SNPs were used as a reference to impute a large set of SNPs with a panel of 301 soybean accessions through whole genome resequencing, GBS and SNP-array (SoySNP50K), and this imputation can be used to fill in missing genotypes and untyped loci with high accuracy^93^. In maize, *CRTRB1, LCYE* and other key genes or genomic regions that govern rate-critical steps in the upstream pathway were identified for various carotenoids using GWAS with 380 CIMMYT inbred lines genotyped by 55 K chip and GBS^94^.

**Figure 3:**
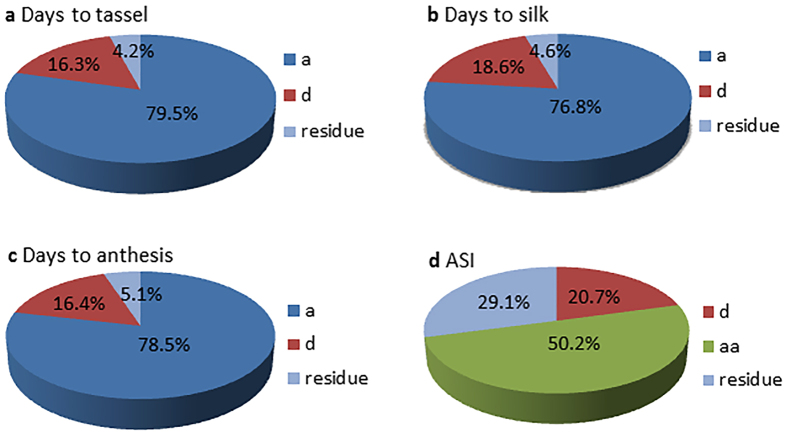
Polygenic component ratios of the flowering traits for maize multiple-hybrid population (MHP). Additive effect accounted for the most of variance for DTT (days to tassel, DTS (days to silk) and DTA (days to anthesis), while additive by additive effect accounted for 50.2% of the variance for ASI (anthesis-silking interval).

**Figure 4:**
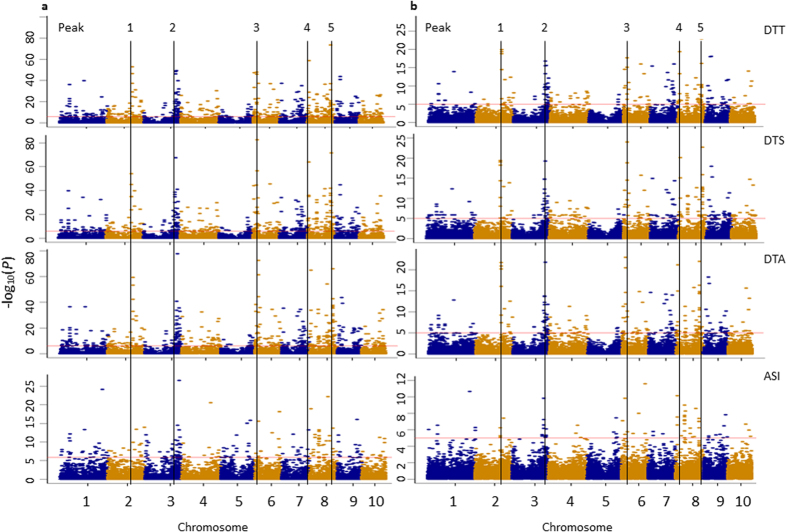
GWAS for four flowering traits with five overlapping peaks identified in the genome using a maize multiple-hybrid population (MHP). (**a**) Manhattan plots for PEPIS. (**b**) Manhattan plots for TASSEL. DTT refers to days to tassel, DTS for days to silk, DTA for days to anthesis and ASI for anthesis-silking interval.

By genotyping the MHP parental lines with the 55 K chip, the genotypes of hybrids can be deduced. In the current MHP, 724 hybrids have been generated by using 51 parental lines, which is much less than what can be generated in all possible ways (51 × (51-1)/2 = 1275). The SNP loci that are heterozygous in one of the two parental lines are scored as missing. SNP markers with missing data rate <20% were extracted to deduce hybrid genotypes. A total of 37,527 SNPs (with minor allele frequency >5% and missing data rate less than 25%) for 724 hybrids were used for structure analysis and GWAS. Polygenic component and main effect (additive and dominant effect) analysis were estimated by a web tool PEPIS (the pipeline for estimating EPIStatic genetic effects, http://bioinfo.noble.org/PolyGenic_QTL/)^95^ (Fig. 3). For PEPIS, three files, including additive genotypic data, dominance data and phenotypic data, were uploaded. The genotype of each hybrid at each specific marker locus was coded as three genotypes, *A* for one homozygous genotype, *B* for the second and *H* for heterozygous genotype. The *K* matrix corresponding to the marker-generated kinship was calculated and used to estimate variance components with restricted maximum likelihood method (REML). The polygenic structure for the trait was examined with the given variance ratios, and genome scanning for main and epistatic effects was performed for each marker (main) effect and marker pair interaction (epistatic) effects^95^. With the sub-pipelines in PEPIS, the sub-pipeline 1 was used to calculate kinship matrix, and sub-pipeline 2 for polygenic component analysis and genome scanning for main and epistatic effect QTL. As a comparison, ‘Q + K’ model for classical GWAS analysis was also performed. Population structure (Q) was determined by fastSTRUCTURE^96^, the number of subpopulation was corrected by neighbor-joining genetic distance^77^, a cluster dendrogram was constructed using FigTree v1.4.2 software (Fig. 2b), and the phenotypic contribution of population structure was estimated with SAS PROC GLM procedure. The kinship matrix (K) and its contribution to phenotype were calculated by TASSEL 5.0 software^97^. The LD (*r*^2^) was calculated to estimate the degree of LD between pairwise SNPs and the sliding window size was set as 500 with a step of 50 markers using TASSEL 5.0^97^ (Fig. 2c). Four flowering traits, DTT (days to tassel), DTS (days to silk), DTA (days to anthesis) and ASI (anthesis-silking interval) are taken as examples for GWAS in this report (Fig. 4). Association analysis were conducted using the compressed mixed linear mixed model (CMLM) and the P3D method including 37,527 SNP markers, population structure (Q), kinship matrix (K) and phenotypic information with TASSEL 5.0 software^97^. The casual genes or QTN associated were identified with a significant threshold (-log_10_ *P* ≥ 5.81) corrected by the Bonferroni test.

Population structure was assessed by fast STRUCTURE for *K* values ranging from 1 to 10, and the population was divided into six subgroups with the correction of NJ genetic distance (Fig. 2b). The phenotypic contribution of population structure for DTT, DTS, DTA and ASI were 0.56, 0.58, 0.58 and 0.03, respectively, and the kinship contribution for phenotype were 0.99, 0.99, 0.98 and 0.52, respectively. The distributions of *r*^2^ over the whole genome were presented in Fig. 2c. The *r*^2^ values declined sharply as the distance increased and the average *r*^2^ was estimated at ∼100 kb, when the cut-off value for *r*^2^ was set to 0.2. The polygenic variance component ratios for the flowering traits are shown in Fig. 3. Additive genetic variance accounted for 79.5%, 76.8% and 78.5% of trait variance for DTT, DTS and DTA, respectively, whereas additive by additive genetic variance accounted for 50.2% variance for ASI, dominant effects accounted secondarily for 16.3%, 18.6%, 16.4% and 20.7%, respectively. Our result is consistent with the previous study for the additive effects for the flowering traits with unimportant epistasis^28^. In PEPIS, polygenic model for additive, dominant, additive by additive, additive by dominant, dominant by additive and dominant by dominant adopted to hybrids and main-effect distribution including additive and dominant are shown in Fig. 4a, and it was confirmed by the results for additive model in TASSEL (Fig. 4b). Therefore, we can run GWAS with hybrid phenotypes using the TASSEL software for additive effect analysis for the traits that are largely controlled by additive genetic variance. Given the additive effect accounted for the most part for the flowering traits and the purpose for this paper is GWAS with maize MHP, dominant effects analysis will be discussed elsewhere.

A large number of significant markers were identified for flowering traits and significant signals around known loci were used to identify candidate genes and QTN co-located with SNPs based on the B73 reference genome (http://www.maizegdb.org/gbowse). Comparing GWAS analyses from PEPIS and TASSEL, we found consistent results for flowering traits with five overlapping peak regions significant for pleiotropy allelic effects for four flowering traits on chromosomes 2, 3, 6 and 8 (Fig. 4). A peak was mapped closed to *GRMZM2G065276* on bin2.06, a homolog of *FCA* on *Arabidopsis* chromosome 4^98^. *Arabidopsis FCA* combined with another gene *FPA* encoded RNA binding protein, downregulated transcription of *FLC*, and promoted flowering^99^. Similarly, two QTN were detected on bin3.07, which were significantly associated with DTS (as *qdsilk1* and *qdsilk8* mapped in a previous research^100^). The related loci *mdh3 (malate dehydrogenase3*) showing four alleles in *Kalmia latifolia*^101^ regulated maize pollen tube growth^102^ and encoded a cytosolic malate dehydrogenase for glyoxylate cycle activity in *Arabidopsis*^103^. The peak on bin6.01 contained *GRMZM2G004959* which had a homologous gene in *Arabidopsis (AT3G61230.1*) expressing in pollen and tube growth^104^. Peak SNPs on the short arm of chromosome 8 (bin 8.01) revealed a phosphatidylethanolamine-binding protein (*ZCN9*) gene *GRMZM2G021614*^105^, the homologous gene in rice *LOC_Os01g02120.1* promoting the transition from vegetative to reproductive growth. The peak mapped on bin 8.05 was predicted with one candidate gene *GRMZM2G091276*, a homologous gene in rice (*LOC_Os05g50890.1) OsGH3.5* coding *JA-amino acid synthetase1* modulating light and JA signal in the photomorphogenesis^106^ and flower opening and anther dehiscence^107^.

For ungenerated hybrids (1275–724 = 551), their performance can be predicted using genomic best linear unbiased prediction (GBLUP)^108^ or empirical Bayesian approach^37^, and in general Bayes and GBLUP possess high accuracy. With an optimized scheme, we should be able to predict performance for much more hybrids with much less hybrids tested. For example, we may be able to predict a full set of diallel hybrids based on the results from NC II mating design using the same set of the parental lines. A statistical method has been developed and applied to such prediction in rice^108^, where a set of 278 selected IMF2 hybrids, developed from a RIL population between Zhenshan 97 and Minghui 63^22^, was used as a training sample to predict the rest 21,667 hybrids, with 16% yield increase in the top 100 selection compared with the average of all hybrids. The results revealed GBLUP as the best method for prediction compared with LASSO and SSVS. The GBLUP involves three stages: construction of respective kinship matrix for training sample and all hybrids, parameter estimation via cross-validation and prediction for missing and rest hybrids^108^. The empirical Bayesian approach was developed and used for a partial NC II mating design in *Brassica napus* including 284 F_1_ hybrids, and 143 restorers and 298 sterile lines were used to predict the phenotypes for missing hybrids, with QTL effects estimated by empirical Bayesian. GCA and SCA would be estimated and elite parents and hybrids could be predicted^37^. In our following research, the GBLUP approach^108^ will be used for analysis of the MHP and reported elsewhere. A recent research showed that predictability of yield was nearly twofold with metabolomics data compared with genomic prediction, despite the latter would be the most efficient method for high-heritability traits^109^, and hybrid prediction by metabolomics data may be a novel pathway in crop breeding programs that should be explored more in the future.

### Breeding using MHPs

Breeding hybrid crops is an important tool that may remarkably promote yield enhancement from 30 to 400% resulting of the heterosis^110^, and hybrid crops were used to investigate heterosis for parental inbreds with maximum combining ability. The populations created should have a higher possibility of acquiring diverse and accurate phenotype performance than natural populations^111^. Diallel and NC mating designs have been widely used in plant breeding programs to maximize selection response and the opportunity for managing coancestry for breeding populations. A prominent feature of diallel and NC mating designs is that both designs provide breeding populations with known pedigrees, which can be used to estimate specific parental genetic effects for backward selection^112^. Estimation of combining ability and heterosis can be used to guide our future breeding activities using the hybrids and their parental lines and derivatives. GCA and SCA, two essential factors in developing breeding strategies, are two main genetic parameters that can be obtained from diallel and NC analysis. GCA reflects the stand or fall of the inbred lines *per se* while SCA is the ability to develop elite hybrids with specific partners. A line × tester population from a collection of 302 diverse inbreds with a common tester B73 was used to predict heterosis by inbred performance and genetic distance between parents for traits, and the result suggested that heterosis could be explained by trait-dependence^113^. An unbalanced NC II for 400 hybrids and 79 inbreds was used for joint analysis of hybrids and parental lines to predict the performance of untested hybrids^114^. A set of NC II crosses between 285 Dent and two Flint inbreds genotyped with 56,110 SNPs and 130 metabolites was used to predict combining ability, allowing a reliable screening of large collections of diverse inbred lines for the potential to create superior hybrids^115^. A set of 136 hybrids and 17 parental lines was used to estimate combining ability and heterosis for stress and non-stress environments^116^, and SCA was significantly correlated with genetic distance. The MHP used in this research contains three subpopulations from temperate, tropical and temperate by tropical hybrids, which can be used for breeding different ecotypes and for gene pyramiding and recombination to combine the merits from different maize germplasm sources.

### Secondary populations derived from the MHP

Many secondary populations can be generated from the parental lines and hybrids of MHP by selfing the hybrids or crossing among parental lines and hybrids. The secondary populations include not only F_2_, BC and RILs, but also MAGIC and NAM. MHP-based researches will provide important information on genetics and genomics such as genes, alleles, haplotypes and population structure about their parental lines and associated hybrids, which can be used to guide further researches on the secondary populations.

As discussed in the previous section, the MHP developed in this study can be shared through distribution of the 51 parental lines, which is much simpler than sharing all possible hybrids (1275) that can be generated from the 51 parents. By sharing the parental lines, a flexible number of hybrids can be generated and tested for agronomic traits of interest to specific users. Genotypic information will be generated and accumulated through genotyping parental lines using different genotyping platforms conducted by collaborators worldwide, which can be shared through a website. As a start point of sharing, we are happy to distribute the 51 parental lines, along with their genotypic information available so far, to those who are interested in using the MHP in their genetics and breeding researches. As a return, we would appreciate all collaborators for sharing their phenotype information generated for specific traits in specific environments or locations and genotypic information generated with their own genotyping platforms.

## Conclusions

Compared with bi- and multi-parental populations and natural ones, MHPs are more suitable for hybrid crops. From various mating designs, a large number of hybrids can be generated to form MHPs, and a set of diallel hybrids from a relatively large number of parental lines would be the best. Each set of diallel hybrids can be divided into three subsets of hybrids with distinct genetic properties, two diallels each representing one ecotype and one NC II representing between-ecotype hybrids. A partial set of hybrids can be generated, selectively or randomly, from the full set, and then used to predict the rest ungenerated hybrids. By sharing parental lines along with their genotypic information, a large number of hybrids can be generated and tested by worldwide collaborators.

In addition to conventional quantitative genetics on combining ability, heterosis and hybrid performance, MHPs can be widely used in modern genetics, genomics and breeding. By high-density genotyping, MHPs can be used in GWAS for phenotypic data collected under diverse environments, including traits of agronomic importance, and heterosis and combining ability as well, which can be based on markers, alleles and haplotypes. MHPs *per se* and their derived secondary populations can be used in breeding for both inbred lines and hybrids. Taking 724 hybrids derived from 51 parental lines and four flowering traits as an example, we compared two independent GWAS methods, PEPIS software developed for hybrids and TASSEL software designed for inbred line populations. The two methods revealed highly consistent results with five overlapping chromosomal regions identified and used for discovery of candidate genes and QTN. Our results indicate that MHPs are powerful in GWAS for hybrid-related traits and will be widely used in the molecular breeding era.

## Additional Information

**How to cite this article**: Wang, H. *et al*. Development of a multiple-hybrid population for genome-wide association studies: theoretical consideration and genetic mapping of flowering traits in maize. *Sci. Rep.* **7**, 40239; doi: 10.1038/srep40239 (2017).

**Publisher's note:** Springer Nature remains neutral with regard to jurisdictional claims in published maps and institutional affiliations.

## Figures and Tables

**Table 1:** 51 maize inbred lines used for development of a multiple-hybrid population (MHP).

No	Name	Pedigree	Origin	Ecotype
P1	NK764	NK235 × B73	USA	Temperate
P2	AS6103	(AS10631 × A632) × (B14AHt × B14AHt)	USA	Temperate
P3	F42	B73 mutation selection	USA	Temperate
P4	FAPW	B14AHt × B37Ht	USA	Temperate
P5	LH51	Mo17 Backcross 5 recovery	USA	Temperate
P6	LX9801	Ye502 × H21	China	Temperate
P7	NS701	A632 × B73Ht	USA	Temperate
P8	PHG83	PH814 × PH207	USA	Temperate
P9	PHN47	Pioneer Hi-Bred proprietary inbred	USA	Temperate
P10	Dan340	Lu Jiu Kuan × C060	China	Temperate
P11	H21	American hybrid P78599	China	Temperate
P12	HZ4	Improved from landrace, Tang Si Ping Tou	China	Temperate
P13	Nan21-3	Jugoslavian Hybrid	China	Temperate
P14	Qi205	(VeiAi141 × ZhongXi017) × Population70	China	Temperate
P15	Ye478	U8112 × Shen5003	China	Temperate
P16	B73	BSSS C5 (Iowa Stiff Stalk Synthetic)	USA	Temperate
P17	LH132	(H93 × B73) × B73	USA	Temperate
P18	Mo17	C103 × 187-2	USA	Temperate
P19	PH4CV	PH7VO × PHBE	USA	Temperate
P20	PH6WC	PH01N × PH09B	USA	Temperate
P21	Chang7-2	HZ4 × WeiChun	China	Temperate
P22	Qi319	American Hybrid P78599	China	Temperate
P23	Si287	444 × 255	China	Temperate
P24	Tie7922	American Hybrid P3382	China	Temperate
P25	Zheng22	(Duqing × E28) × Lu JiuKuan	China	Temperate
P26	Zheng58	Variant plants from Ye478	China	Temperate
P27	Dan598	(Dan340 × Danhuang11) × (Danhuang02 × Dan599)	China	Temperate
P28	Huang C	(Huangxiao162 × Zi330/02) × Tuxpeno	China	Temperate
P29	TR0397	[CML159/[CML159/[MSRXPOOL9]C1F2-205-1(OSU23i)-5-3-X-X-1-BB]F2-3sx]-8-1-1-BB-1-B	CIMMYT	Tropical
P30	CL04934A	CL-04934 (P49C2H12-5-4xP23C2-11-1)-2-2-2-B*10	CIMMYT	Tropical
P31	CML418	G15C22H100#-2-2-6-1-2-BBBBB	CIMMYT	Tropical
P32	CML84	G32C19H512-2-#1-B-####-6-B	CIMMYT	Tropical
P33	CML96	P42C4F233-1-#2-B-#*6-2-B	CIMMYT	Tropical
P34	Chuan29 Female	Commercialized hybrid	China	Tropical
P35	TR0395	[(SML × SMQPM) × (MTL × SMQPM)]F1S6-1-25-BB-1-B	CIMMYT	Tropical
P36	TR0403	[CML389/CML176]-B-29-2-2-B*5	CIMMYT	Tropical
P37	TR0412	[DTPWC8F31-4-2-1-6/CML444//ZM521B-66-4-1-1-1-BB]-3-2-1-B-B-B	CIMMYT	Tropical
P38	TR0415	[Ent320:92SEW2-77/[DMRESR-W]EarlySel-#I-2-4-B/CML386]-B-11-3-B-2-#-B*4	CIMMYT	Tropical
P39	TR0423	02SADVL2B-#-16-2-1-B-B-B	CIMMYT	Tropical
P40	18-599	American Hybrid P78599	China	Tropical
P41	622016-ZCN-2	(Zhongnuo No.2 × H99) × H99 × H99	CIMMYT	Tropical
P42	CML206	[EV7992#/EVPO44SRBC3]#BF37SR-2-3SR-2-4-3-BB	CIMMYT	Tropical
P43	CML312SR	MAS[MSR/312]-117-2-2-1-B*4-B-B-B-B	CIMMYT	Tropical
P44	CML312SRQ	CML312SRQ = [[(CLQ-RCWQ83xCML312SR)xCML312SR]xCML312SR)]-15-1-BBB	CIMMYT	Tropical
P45	CML330	89[SUWAN8422]/[P47S3/MP78:518]#-7-1-1-1-1-1-B-#-BB	CIMMYT	Tropical
P46	CML504	[COMPE2/P43SR//COMPE2]F#-20-1-1-B-1-BB-6-BB	CIMMYT	Tropical
P47	CML85	P34C5F21-2-#1-2-2-#	CIMMYT	Tropical
P48	DTMA227B	DTPWC9-F104-5-4-1-1-B-B-B	CIMMYT	Tropical
P49	TR0501	INTA-191-2-1-2-B*8-B-B-B	CIMMYT	Tropical
P50	TR0582	P501SRc0-F2-47-3-1-1-B-B-B-B	CIMMYT	Tropical
P51	Jiao51	Improved from landrace	China	Tropical

**Table 2:** Key Chinese inbreds used in a multiple-hybrid population (MHP)and their contribution to hybrid and inbred breeding programs.

Inbreds	Characteristics	Hybrids released	Inbreds developed	Hybrids released with derivatives
LX9801	Compact plant type, high combining ability, strong resistance to NCLB, SCLB, MDMV and head smut	Ludan981, Ludan984	LX03-2, Jing92	Jingke968, Ludan9066
Chang7-2	Compact plant type, small tassel size, less branch number, abundant pollen and high GCA	Anyu5, Zhengdan958, Jidan7, Jifeng96	Xun926, Xun92-8, K17, HD568	Xundan20, Zhongdan909
Dan340	Erect leaves above ear, flourishing roots, strong resistance to NCLB and head smut	Yedan13, Danyu15, Jidan159 and Jidan27	Tie9010, Xi502, Dan232	Xiyu3, Yedan20, Tiedan12, Dan605
H21	Broad leaf blade, long internode, small ASI, strong resistance to NCLB, head smut and stalk rot	Jindan33, Xinkang5	LX9801, Ningchen07, M81	Ningyu309, Zhongxing56, Ludan999
HZ4	High combining ability, strong disease resistance, high seed yield, compact plant type	Yandan14, Yedan2, Yedan4	Chang7-2, Ji444, K12, 897, Ji854, Tianya4, Ji856, 196, Ji853	Liaoyu22, Sidan104, Jidan180, Shandan911
Nan21-3	Flourishing roots, stiff stalk, small ASI, strong resistance to NCLB and dwarf mosaic disease	Nansandanjiao, Nanqidanjiao, Nanqisanjiao, Nanhuangdanjiao	286-4, Bai286	Wandan11, Ke’en18, Wandan14
Qi205	High combining ability, strong resistance, high seed yield and good adaptability	Chengdan17, Chengdan18, Zhongdan9409	Qi3925, ISA01	Ludan718, Kexiang11
Qi319	Compact-type plant, abundant pollen, strong resistance to lodging and southern corn rust	Ludan50, Ludan963	LX3999, JN15, PC58	Ludan6006, Jingke308, Tiantai33
Si287	Slender leaves, abundant pollen, high seed-setting rate, and high combining ability	Jidan27, Jidan46	KM36	Jinongda115, Jinongda578
Tie7922	Stiff stem, strong resistance to drought, lodging and waterlogging, high resistance to NCLB and SCLB, head smut and stalk rot	Tiedan8, Yayu2, Simi25	TieC8605-2, Dan9046, Liao2345	Tiedan10, Tiedan19, Danyu2, Danyu26, Liaodan24
Ye478	High combining ability, compact-type, short and stout stem, good comprehensive agronomic traits	Zhongdan8, Yedan12, Yedan13, Yedan19	K22, Jing89, Yuqing858	Shandan21, Shandan308, Jingyu16, Yuqing1
Zheng22	Stocky stem, superior root system, strong resistance to drought, NCLB and SCLB, MRDV and BYDV	Yuyu18	Shen151, Zheng22you	Shenyu17, Shenyu20, Wuke1
Zheng58	High combining ability, compact plant type, strong resistance to NCLB, SCLB and lodging	Ludan9002	M6, WK858	Huanong18, Weike702
Dan598	Good stems elasticity and strong resistance to BLSB and head smut	Danyu26, Danyu39, Danke2151, Dongdan60	M5972, Y14, 765	Liangyu99, Huatian866, Danyu502
Huang C	Compact plant type, high lysine content, strong resistance to NCLB and SCLB	Nongda108, Liyu10	168, T14-3B	Changyu1, Jindan66, Zhongyu335
Jiao51	High combining ability, good grain quality, stiff stem and lodging resistance	Jiaosandanjiao, Andan136, Yudan11, Yayu15		
18-599	High combining ability, high seed yield, good quality and good comprehensive agronomic traits	Shengyu9, Dong315, Chunxi11	Zhongyu335, Yayu318	YA0419
Chuan 29 Female	High combining ability and heterosis, high ferric content	Chuandan29		

NCLB: Northern corn leaf blight; SCLB: Southern corn leaf blight; MDMV: Maize dwarf mosaic virus; MRDV: Maize rough dwarf virus; BYDV: Barley yellow dwarf virus; BLSB: Banded leaf sheath blight; ASI: anthesis-silking interval.

**Table 3:** Agronomic traits observed for parental lines and hybrids in a multiple-hybrid population (MHP).

Trait	Temperate inbreds	Tropical inbreds	Temperate diallel	Tropical diallel	NC II
Plant height	182.73^C^(20.88)	183.36^C^(14.94)	252.56^AB^(19.85)	247.85^B^(11.37)	269.73^A^(14.87)
Ear height	74.24^D^(14.78)	90.26^C^(15.98)	111.13^B^(14.60)	116.25^AB^(9.20)	131.86^A^(13.78)
Days to tassel	56.41^B^(3.26)	65.59^A^(3.82)	51.89^C^(2.07)	58.50^B^(2.82)	56.81^B^(2.28)
Days to silk	61.39^B^(3.14)	70.21^A^(4.23)	55.89^C^(1.89)	62.30^B^(3.26)	60.86^B^(2.39)
Days to anthesis	59.53^B^(3.22)	68.80^A^(4.03)	54.16^C^(1.91)	60.90^B^(3.03)	59.05^B^(2.36)
Anthesis-silking interval	2.19^AB^(0.33)	2.55^A^(0.28)	1.68^B^(0.33)	1.60^B^(0.53)	1.87^AB^(0.35)
Ear length	13.66^C^(1.43)	12.45^D^(1.28)	17.83^A^(1.42)	15.65^B^(0.53)	17.96^A^(1.02)
Ear diameter	38.91^B^(2.84)	35.08^B^(2.19)	46.16^A^(2.34)	46.15^A^(1.66)	46.85^A^(2.35)
Row number	13.68^C^(1.43)	12.04^D^(0.89)	15.62^A^(1.20)	14.35^BC^(1.02)	15.02^AB^(1.38)
Grain number per row	21.66^B^(2.42)	18.68^B^(1.77)	35.50^A^(3.03)	33.00^A^(1.45)	34.78^A^(2.70)
Grain number	302.76^C^(45.01)	237.41^C^(25.18)	554.37^A^(56.91)	474.25^BC^(42.90)	522.45^AB^(53.96)
Hundred grain weight	26.56^BC^(3.43)	23.63^C^(3.04)	29.32^AB^(3.26)	31.45^A^(1.85)	33.13^A^(3.08)
Grain weight per plant	52.91^B^(10.23)	44.77^B^(6.38)	128.90^A^(18.96)	135.35^A^(3.91)	134.70^A^(8.64)

Different letters in the same row indicate significant difference by Duncan test at 1% probability, and standard errors are given in parenthesis.

**Table 4:** Agronomic traits for the hybrids from parental inbreds in different heterotic groups.

Traits	Lancaster	PA	PB	Pioneer	Reid	LRC	SPT
Plant height	256.55^B^(21.10)	255.23^BC^(15.01)	264.32^A^(13.77)	252.22^CD^(19.66)	244.84^E^(19.71)	248.64^D^(15.91)	260.81^A^(20.59)
Ear height	110.44^BC^(13.80)	113.89^B^(10.79)	119.63^A^(10.61)	107.37^CD^(11.56)	106.90^CD^(14.52)	106.30^D^(12.07)	120.02^A^(17.12)
Days to tassel	52.09^C^(1.98)	52.74^B^(1.85)	56.39^A^(1.82)	51.36^DE^(2.00)	51.25^E^(2.04)	51.75^CD^(1.67)	52.07^C^(2.28)
Days to silk	56.06^CD^(1.72)	56.83^B^(1.76)	57.64^A^(1.44)	55.23^E^(1.63)	55.50^E^(1.98)	56.15^C^(1.69)	55.64^DE^(1.95)
Days to anthesis	54.42^C^(1.79)	54.99^B^(1.77)	55.76^A^(1.79)	53.52^D^(1.73)	53.61^D^(1.91)	54.17^C^(1.65)	54.25^C^(2.09)
Anthesis-silking interval	1.63^AB^(0.41)	1.76^A^(0.35)	1.80^A^(0.38)	1.66^AB^(0.37)	1.82^A^(0.39)	1.78^A^(0.38)	1.40^B^(0.32)
Ear length	18.53^B^(1.45)	17.90^C^(1.06)	19.80^A^(0.93)	17.69^CD^(1.28)	17.38^D^(1.35)	18.47^B^(1.10)	17.62^CD^(1.45)
Ear diameter	45.68^B^(2.46)	46.68^AB^(1.74)	47.90^A^(1.80)	45.45^B^(2.46)	45.37^B^(2.24)	46.62^AB^(2.34)	47.87^A^(2.12)
Row number	15.18^CD^(1.01)	15.32^C^(0.98)	15.06^D^(0.92)	15.97^A^(1.11)	15.76^B^(1.07)	16.05^A^(1.19)	15.75^B^(1.10)
Grain number per row	37.83^A^(3.76)	35.72^C^(2.20)	37.15^B^(2.34)	34.46^D^(2.86)	34.65^D^(3.27)	36.52^B^(2.38)	35.52^C^(2.73)
Grain number	573.54^AB^(64.56)	546.88^C^(44.00)	558.86^C^(45.52)	550.13^C^(55.82)	546.96^C^(61.95)	584.17^A^(59.83)	560.15^BC^(53.57)
Hundred grain weight	29.29^CD^(3.18)	30.19^BC^(2.49)	33.29^A^(1.62)	28.77^CD^(3.90)	28.04^D^(3.14)	28.26^D^(3.00)	30.94^B^(2.73)
Grain weight per plant	130.78^BC^(20.94)	129.09^BC^(15.34)	154.90^A^(13.17)	124.96^BC^(20.98)	120.03^C^(21.47)	130.73^BC^(16.98)	141.00^AB^(16.14)

Different letters in the same row indicate significant difference by Duncan test at 1% probability, and standard errors are given in parenthesis.
